# Impedance Spectroscopy Analysis of Thermoelectric Modules Fabricated with Metallic Outer External Layers

**DOI:** 10.1021/acsaelm.1c00670

**Published:** 2021-11-12

**Authors:** Braulio Beltrán-Pitarch, Jorge García-Cañadas

**Affiliations:** Department of Industrial Systems Engineering and Design, Universitat Jaume I, Campus del Riu Sec, Castelló de la Plana 12006, Spain

**Keywords:** Peltier device, frequency domain, thermal contact conductance, thermal interface, metallic layer

## Abstract

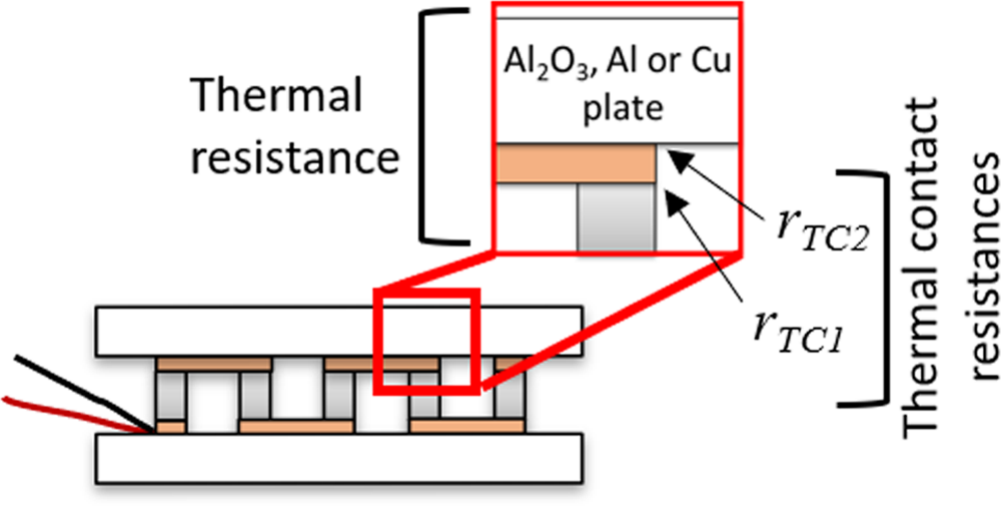

In recent years, thermoelectric (TE) devices have been used in several refrigeration applications and have gained attention for energy generation. To continue the development of devices with higher efficiency, it is necessary not only to characterize their materials but also to optimize device parameters (e.g., thermal contacts). One attempt to increase the efficiency at the device level consists of the replacement of the typical ceramic layers in TE modules by metallic plates, which have higher thermal conductivity. However, this alternative device design requires the use of a very thin electrical insulating layer between the metallic strips that connect the TE legs and the outer external layers, which introduces an additional thermal resistance. Impedance spectroscopy has been proved to be useful to achieve a detailed characterization of TE modules, being even capable to determine the internal thermal contact resistances of the device. For this reason, we use here the impedance method to analyze the device physics of these TE modules with outer metallic plates. We show for the first time that the impedance technique is able to quantify the thermal contact resistances between the metallic strips and the outer layers, which is very challenging for other techniques. Finally, we discuss from our analysis the prospects of using TE modules with external metallic plates.

## Introduction

Thermoelectric (TE) materials have the ability to convert heat into electricity or use electricity to create a temperature difference. In recent years, they have been used in several refrigeration applications, such as electronic device cooling and room-temperature cooling, and have gained attention in various energy harvesting fields such as industrial waste heat, power generators in aerospace, and energy efficient vehicles.^1,2^ The conversion efficiency of a TE system not only depends on the properties of the TE materials, but also on the temperature difference between their edges. Hence, one strategy to maximize efficiency is to maintain the temperature at the edges of the TE materials as similar as possible to the temperature of the heat source and heat sink. In typical TE modules, the thermal resistance from the edges of the TE materials to the outer faces of the TE device is formed by (i) the thermal contact resistance^3^ (and the spreading-constriction resistance^4^ due to the change in area) between the TE legs and the metallic strips that connect the legs, (ii) the thermal resistance of the metallic strips, (iii) the thermal contact (and spreading-constriction) resistance between the metallic strips and the outer ceramics, and (iv) the thermal resistance of the ceramics. The use of external metallic layers replacing the standard ceramic plates can serve to reduce the thermal resistance (and the spreading-constriction resistance) of the external layers of the device, due to the usually high thermal conductivity of metals. However, in this alternative device design the metallic layers are also electrically conductive, so it is necessary to introduce an additional thin insulating layer between the metallic strips and the external layers, which may increase the thermal contact resistance at such interface.

Impedance spectroscopy is a powerful technique to understand device physics and it is used in many scientific fields.^5^ Since the idea of using impedance spectroscopy for the characterization of TE materials and devices was born,^6,7^ it has been proved to be useful for the determination of the figure of merit *zT*,^8−11^ or even to perform a complete characterization of TE modules (determination of the ohmic resistance, the average Seebeck coefficient and thermal conductivity of the TE legs, and *zT*) in suspended conditions if the thermal conductivity of the ceramics is known.^12−17^ More recently, we have shown how to characterize the thermal contact resistance between the TE legs and the metallic strips.^18^ In the latter study, we developed the most comprehensive equivalent circuit to date, which includes, among other key phenomena, the thermal contact resistance between the metallic strips and the outer layer. This parameter is essential to evaluate the effect of the thin insulating layers in modules with external metallic layers.

Hence, we make use here of our recently developed impedance equivalent circuit^18^ to perform fittings to TE modules with aluminum and copper outer layers. The fitting of experimental data to an impedance equivalent circuit is the most common way adopted to extract the parameters of interest from the system under study. These alternatively designed modules use a thin epoxy layer between the metallic strips and the outer metallic layers to avoid the electrical contact, which introduces thermal contact resistances. We show for the first time how impedance spectroscopy can characterize these thermal contacts, which is very difficult to achieve by other techniques. The results are compared with a TE module with the typical insulating ceramic plates, which, as expected, does not show any thermal contact at those interfaces but introduces a larger thermal resistance from the outer layer materials themselves (and spreading-constriction) because of their lower thermal conductivity. After performing the impedance analysis, we discuss the prospects of using TE modules with metallic external layers based on the thermal resistance of each type of module.

## Equivalent Circuit

**Figure 1:**
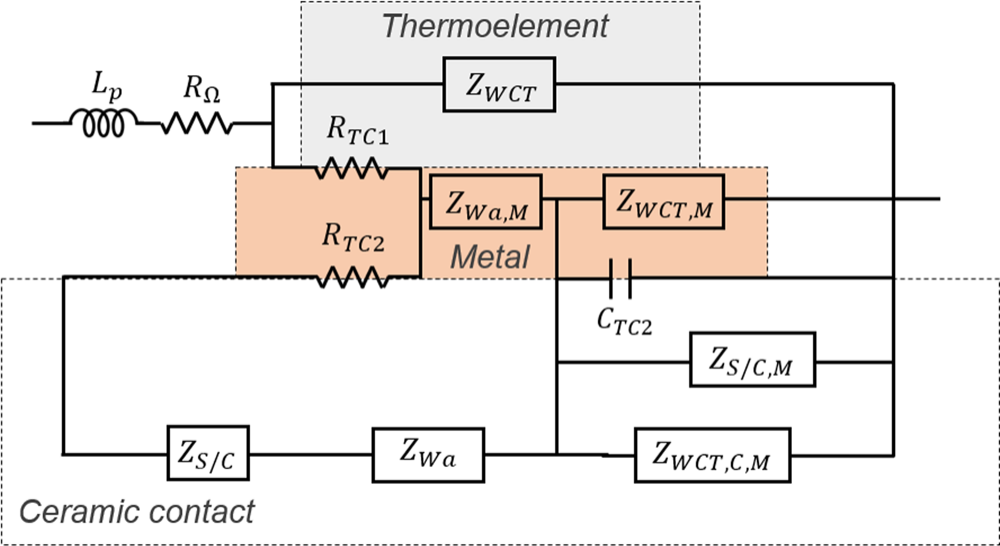
Simplified equivalent circuit of a suspended thermoelectric device when convection and radiation effects are negligible. Reprinted from ref (18). License under CC BY 4.0.

**Figure 2:**
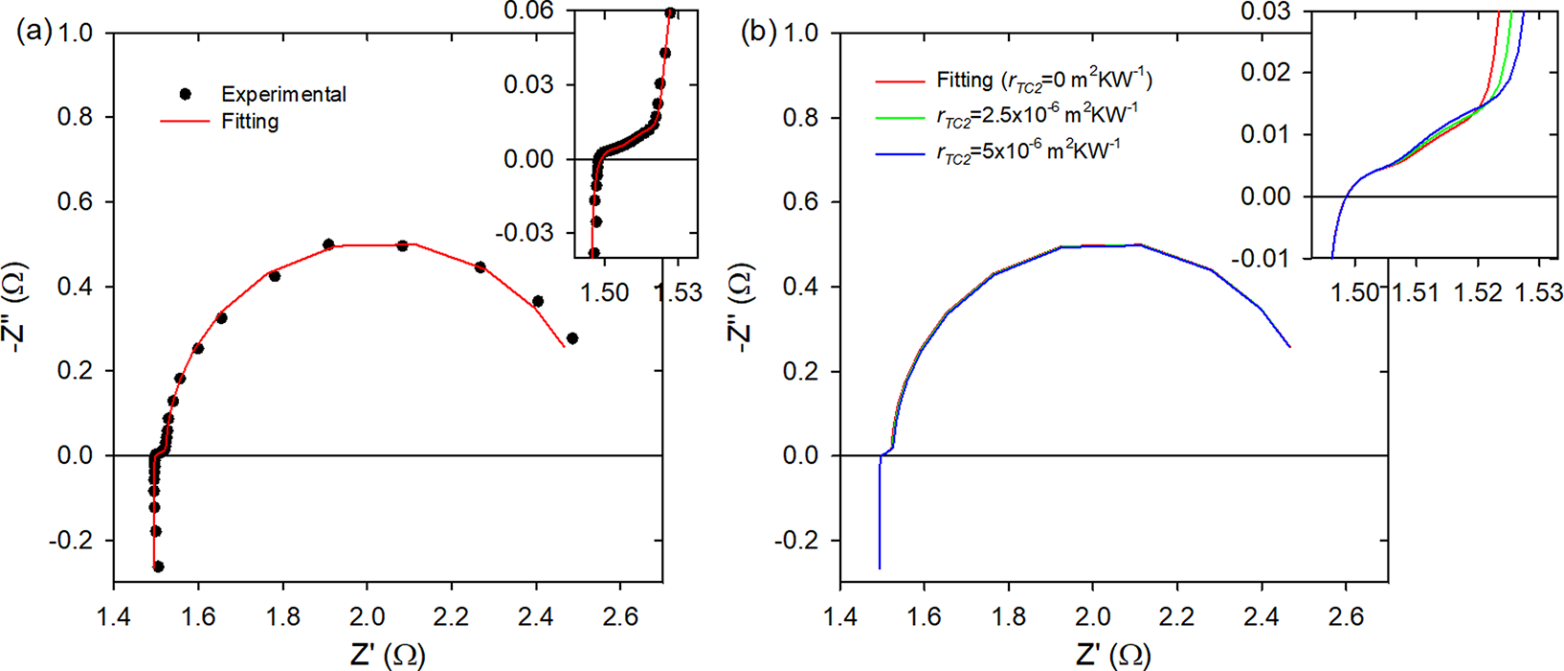
(a) Experimental impedance spectroscopy measurement of module-alumina (dots) and its fitting (line). (b) Impedance spectroscopy simulations using the parameters of the fitting in panel a and varying *r*_TC2_. The insets show a magnification of the high-frequency part.

The equivalent circuit used in this study (see Figure 1) is the one shown in Figure 2b of our recently published work,^18^ which neglects convection and radiation effects and considers the module suspended in vacuum. The equivalent circuit was developed considering 2*N* (*N* being the number of TE couples) cylindrical legs with area *A* and length *L* in contact with metallic strips of area *A*/*η*_M_ (where *η*_M_ is the ratio between the area of all the TE legs and the area of all the metallic strips) and length *L*_M_, which are also in contact with the outer external layers of area *A*/*η* (where *η* is the ratio between the area of all the TE legs and the external layers, also known as filling factor) and length *L*_C_. The spreading-constriction resistance between the TE legs and the metallic strips is neglected, but it is considered between the metallic strips and the external layers. In addition, the thermal contact resistivities between the TE legs and the metallic strips *r*_TC1_ and between the metallic strips and the external layers *r*_TC2_ were introduced.

The impedance function that defines the equivalent circuit of Figure 1 and, hence, was used to perform the fittings to the experimental measurements in this study is
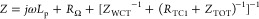
1where *j* = (−1)^0.5^ is the imaginary number, ω the angular frequency (ω = 2π*f*, being *f* the frequency), *R*_Ω_ the total ohmic resistance of the TE device, *L*_p_ the parasitic inductance,^19^ and the impedance *Z*_TOT_ is defined as
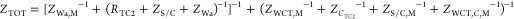
2The elements in eqs 1 and 2 are defined by
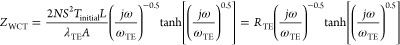
3
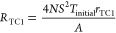
4
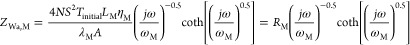
5
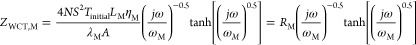
6
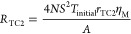
7
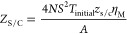
8
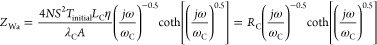
9
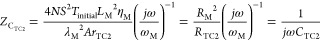
10
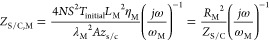
11
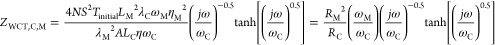
12where *S* is the average Seebeck coefficient of all the TE legs, *T*_initial_ is the ambient temperature, and *λ_i_*, *α*_*i*_, and *ω*_*i*_ are the average thermal conductivity, thermal diffusivity and characteristic angular frequency of each material, respectively: TE legs [*i* = TE, *ω*_TE_ = *α*_TE_/(*L*/2)^2^], metallic strips (*i* = *M*, *ω*_M_ = *α*_M_/*L*_M_^2^), and external layers (*i* = *C*, *ω*_C_ = *α*_C_/*L*_C_^2^). Finally, the spreading-constriction impedance is defined as
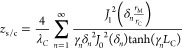
13with *J*_0_ and *J*_1_ being the first kind Bessel functions of order zero and one, respectively, *r*_M_ and *r*_C_ the equivalent radii of the metallic strips and outer external layers, respectively, *δ*_*n*_ is the *n*th zero of *J*_1_, and *γ*_*n*_ is the value for each *δ*_*n*_ that verifies
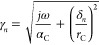
14When ω → 0, eq 13 becomes the spreading-constriction resistance, which is defined as
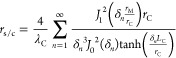
15and is needed to compare the benefits of using an external metallic layer.

It should be noticed that when a fitting to an experimental impedance spectroscopy measurement is performed with this equivalent circuit, the thermal contact resistivities *r*_TC1_ and *r*_TC2_ are directly obtained, which is very challenging for other techniques.

## Results and Discussion

Three different TE modules provided by Jeongkwan Co. Ltd. were used in this study: module-alumina, module-Cu, and module-Al. All of them are modules of 40 mm × 40 mm with *N* = 127, *L* = 1.6 mm, *L*_M_ = 0.2 mm, *A* = 1.73 mm × 1.73 mm, *η*_M_ = 0.87, and η = 0.48. The difference between the three modules lies in their outer layers. Although module-alumina uses the typical alumina insulating plates with *L*_C_ = 1.0 mm, module-Cu and module-Al use copper and aluminum plates of *L*_C_ = 0.2 mm, respectively (electrically insulated with an epoxy layer of around 0.05 mm). As the thickness of this insulating layer is very small, the heat that they can accumulate can be neglected, and their presence only introduces a thermal contact resistivity *r*_TC2_.

Impedance spectroscopy measurement were performed to the different modules. An *I*_ac_ = 30 mA was used after its optimization as described in ref (20). Amplitude optimization basically consists in identifying the lowest possible current amplitude (*I*_ac_) without noise in the spectra. The frequency range from 10 mHz to 1 MHz and 50 measuring points (logarithmically distributed in the frequency range) were chosen to ensure a proper number of points in the regions of interest in the spectra, mainly in the high frequency part. All the measurements were performed inside a vacuum chamber with the modules suspended from their cables under vacuum (<5 × 10^–4^ mbar) and at room temperature with a PGSTAT302N potentiostat (Metrohm Autolab B. V.) equipped with a FRA32 M impedance module.

**Figure 3:**
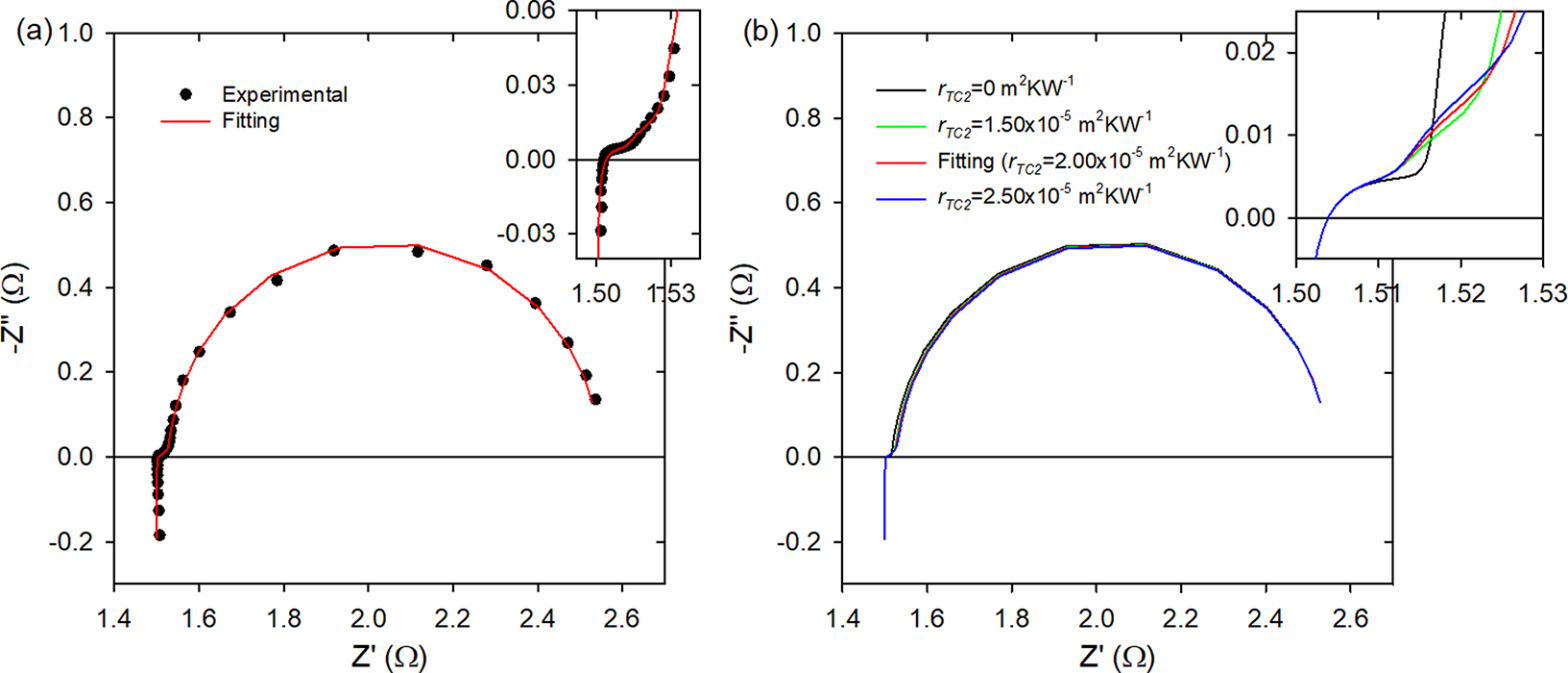
(a) Experimental impedance spectroscopy measurement of module-Cu (dots) and its fitting (line). (b) Impedance spectroscopy simulations using the parameters of the fitting in panel a and varying *r*_TC2_. The insets show a magnification of the high-frequency part.

**Figure 4:**
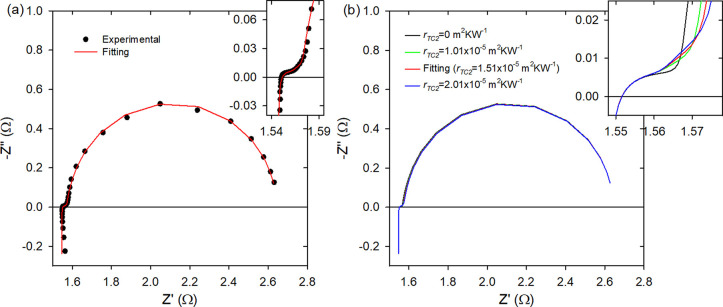
(a) Experimental impedance spectroscopy measurement of module-Al (dots) and its fitting (line). (b) Impedance spectroscopy simulations using the parameters of the fitting in panel a and varying *r*_TC2_. The insets show a magnification of the high-frequency part.

Figures 2a, 3a, and 4a show the experimental impedance spectrum (dots) and its fitting using the equivalent circuit of Figure 1 (lines) of module-alumina, module-Cu, and module-Al, respectively. The fittings were performed using the *Matlab* code provided in the Supporting Information of ref (18). It should be noted that six points at the highest frequencies were not included in the fitting because they deviate from a purely inductive behavior. The fittings were obtained using the procedure recommended in the mentioned article. First, *L*_p_, *R*_Ω_, *r*_TC1_, *r*_TC2_, *λ*_TE_, and *λ*_C_ are fitted, maintaining fixed *S*, *α*_TE_, *α*_C_, *λ*_M_, and *α*_M_. We provided the fixed values of *α*_TE_ = 0.37 mm^2^ s^–1^, *λ*_M_ = 400 W m^–1^ K^–1^, *α*_M_ = 110 mm^2^ s^–1^, *α*_C_ = 10 mm^2^ s^–1^ for module-alumina, *α*_C_ = *α*_M_ for module-Cu, and *α*_C_ = 90 mm^2^ s^–1^ for module-Al.

Fixed values of the Seebeck coefficients were also provided from their direct measurement, which resulted in values of 190.08, 193.03, and 191.14 μVK^–1^, for module-alumina, module-Cu, and module-Al, respectively. The Seebeck coefficient was measured by applying different constant currents (20, 40, 60, 80, 100, and 120 mA) to the TE modules suspended in vacuum (<5 × 10–4 mbar). Once steady state is achieved after applying each current value, a temperature difference appears that is due to the Peltier effect. After reaching the steady state, the circuit was opened and the voltage and the temperature difference between the outer layers were measured immediately. The temperature difference was measured with thermocouples touching the central part of the outer layers and using a bit of thermal grease at their tips. The Seebeck coefficient was obtained from the slope of the voltage vs temperature difference plot.

Then, when the error in rTC2 was >100% and its value in the order of 1 × 10^–7^ m^2^ KW^–1^, which indicates that this thermal contact resistance can be neglected, a second fitting with *r*_*TC2*_ = 0 was performed. This was the case for module-alumina, because the thermal contacts between the metallic strips and the alumina layers are usually good.^21^ However, it was not necessary for module-Cu and module-Al, as the thin electrical insulating layer between the metallic strips and the outer metallic layers introduced a thermal contact resistance at these junctions. Consequently, a single fitting was performed to module-Cu and module-Al, which provided both *r*_TC1_ and *r*_TC2_ (see Table 1). It should be noticed that the fitting errors of *r*_TC2_ were somewhat high, as can be seen in Table 1. These errors provide an indication of the deviation of the parameters provided by the fitting with respect to the experimental points. It should be noted that they do not represent the total errors, as other sources of error could contribute, e.g., the errors in the measured Seebeck coefficient and the assumed thermal diffusivities.

**Table 1:** Fitting Parameters with Their Associated Relative Errors (in brackets) Obtained for the Three TE Modules Used in This Work^a^

name	*L*_p_ (H)	*R*_Ω_ (Ω)	*r*_TC1_ (m^2^ KW^–1^)	*r*_TC2_ (m^2^ KW^–1^)	*λ*_TE_ (W m^–1^ K^–1^)	*λ*_C_ (W m^–1^ K^–1^)
module-alumina	4.06 × 10^–7^	1.49	1.17 × 10^–5^		1.41	26.93
	(1.85%)	(0.037%)	(6.37%)		(0.79%)	(1.03%)
module-Cu	2.92 × 10^–7^	1.50	1.13 × 10^–5^	2.00 × 10^–5^	1.45	466.5
	(1.55%)	(0.029%)	(7.67%)	(12.79%)	(0.49%)	(1.03%)
module-Al	3.61 × 10^–7^	1.55	1.47 × 10^–5^	1.51 × 10^–5^	1.36	277.6
	(1.40%)	(0.030%)	(7.20%)	(24.30%)	(0.44%)	(1.65%)

a The fittings were performed with the *Matlab* code provided in the Supporting Information of ref (18).

Figures 2b, 3b, and 4b show simulations that include the spectrum from the fitting to the experimental data of module-alumina, module-Cu, and module-Al, respectively, and spectra simulated using the fitting parameters but varying *r*_TC2_. The insets show the magnification of the high frequency part, where the changes in the impedance spectra are more prominent.

For module-alumina, the inset of Figure 2b shows that a higher value of *r*_TC2_ produces a larger linear part of the impedance spectra and introduces a curvature in this region, which is not observed experimentally (see the inset of Figure 2a). This behavior was not surprising, because this TE module does not contain the thin insulating epoxy layer but the usual insulating ceramic layers.

Module-Cu shows a higher slope in the straight-line region of the impedance spectrum, whose size and slope depend on *r*_TC2_. If *r*_TC2_ was not present, the straight-line region would vanish altogether (see the inset of Figure 3b) because of the high thermal conductivity of the Cu external layers.

Finally, module-Al shows features at high frequency similar to those of module-Cu, as the thermal conductivity of the aluminum outer layers is also high. In this case, *r*_TC2_ is slightly lower than in module-Cu (see Table 1), which reduces the size and the curvature of the straight zone.

The results obtained above by means of the impedance method, allow the comparison of the TE modules with external metallic layers (module-Cu and module-Al) with the standard ceramic plates configuration (module-alumina). For this purpose, the thermal resistance due to the presence of *r*_TC2_, *r*_S/C_, and the resistance of the outer layer itself was calculated (see Table 2). The table also includes in brackets the thermal resistances for module-Cu and module-Al if it is assumed that they have the same length of the outer layer of module-alumina (*L*_C_ = 1 mm). It can be seen in Table 2 that the main contributor to the total thermal resistance in module-alumina is the conduction in the outer layer, which is a consequence of its low thermal conductivity, and for module-Cu and module-Al, is the thermal contact resistivity *r*_TC2_. It can also be seen that *r*_S/C_ has a higher contribution in module-alumina, as it is inversely proportional to the thermal conductivity of the outer layer (see eq 15). Finally, it is interesting to compare the addition of all the thermal resistances, which shows that the lowest thermal resistance is obtained for module-Al (due to its lower *r*_TC2_), followed by module-Cu, and then by module-alumina. These results show how the use of outer metallic layers may reduce the total thermal resistance between the TE legs and the heat source/sink.

**Table 2:** Thermal Resistances Due to (i) the Contacts between Metallic Strips and Outer Layers, (ii) Spreading-Constriction at the Same Location, and (iii) Outer Layers for the Three TE modules Analyzed in This Study.^a^

name	*r*_TC2_*η*_M_*A*^–1^ (KW^–1^)	*r*_S/C_*η*_M_*A*^–1^ (KW^–1^)	*L*_C_*ηA*^–1^*λ*_C_^–1^ (KW^–1^)	(*r*_TC2_ + *r*_S/C_)*η*_M_*A*^–1^ + *L*_C_*ηA*^–1^*λ*_C_^–1^ (KW^–1^)
module-alumina	0	1.33	5.96	7.29
module-Cu	5.81	0.14 (0.08)	0.07 (0.34)	6.02 (6.23)
module-Al	4.39	0.24 (0.13)	0.12 (0.58)	4.74 (5.10)

a The final column shows the addition of the three values. The values for module-Cu and module-Al if LC = 1 mm, as is the case for module-alumina, are given in parentheses..

It is also interesting to remark that the fabrication of TE modules with outer metallic layers and epoxy insulation may not only be more beneficial to reduce the total thermal resistance inside the TE devices but also reduce it at a system level. For example, the metallic strips that connect the TE legs may directly be attached to the heat source/sink (with the epoxy insulation), and hence, one thermal interface is removed altogether, which can significantly benefit the performance.

## Conclusions

Our recently developed comprehensive equivalent circuit, which includes the internal thermal contact resistances in TE modules, was used to fit TE modules with metallic outer external layers. These modules need a thin insulating layer (epoxy) between the metallic contacts that connects the TE legs and the external metallic layer, which introduces a thermal contact resistance in that interface. We showed how impedance spectroscopy is capable of quantifying these thermal contact resistances, which are usually negligible in TE modules with the typical ceramic layers, by measuring two modules with outer metallic layers (copper and aluminum). The insulating epoxy layer introduced a thermal contact resistivity of 2.00 × 10^–5^ and 1.51 × 10^–5^ m^2^ KW^–1^ for the copper and aluminum modules, respectively. The use of metallic layers not only reduces the thermal resistance of the outer layer but also reduces the spreading-constriction resistance, which was enough to compensate for the additional thermal contact resistance added by the epoxy layer. The lowest total thermal resistance was obtained with the use of aluminum, followed by copper, and finally the typical ceramic. Even though only one TE module of each type was measured, which is not enough to determine the uncertainty of the thermal contact resistance introduced by the epoxy layers, these results are very promising for the development of TE modules without ceramics.
